# Current insights into genome-based personalized nutrition technology: a patent review

**DOI:** 10.3389/fnut.2024.1346144

**Published:** 2024-01-22

**Authors:** Soo-Hyun Park, Hyo-Kyoung Choi, Jae Ho Park, Jin-Taek Hwang

**Affiliations:** Food Functionality Research Division, Korea Food Research Institute, Wanju-gun, Jeollabuk-do, Republic of Korea

**Keywords:** patent, personalized nutrition, genomics, disease, prevention

## Abstract

Unlike general nutritional ranges that meet the nutritional needs essential for maintaining the life of an entire population, personalized nutrition is characterised by maintaining health through providing customized nutrition according to individuals’ lifestyles or genetic characteristics. The development of technology and services for personalized nutrition is increasing, owing to the acquisition of knowledge about the differences in nutritional requirements according to the diversity of individuals and an increase in health interest. Regarding genetics, technology is being developed to distinguish the various characteristics of individuals and provide customized nutrition. Therefore, to understand the current state of personalized nutrition technology, understanding genomics is necessary to acquire information on nutrition research based on genomics. We reviewed patents related to personalized nutrition-targeting genomics and examined their mechanisms of action. Using the patent database, we searched 694 patents on nutritional genomics and extracted 561 highly relevant valid data points. Furthermore, an in-depth review was conducted by selecting core patents related to genome-based personalized nutrition technology. A marked increase was observed in personalized nutrition technologies using methods such as genetic scoring and disease-specific dietary recommendations.

## 1 Introduction

The general scope of nutrition is the study of the requirements or characteristics of nutrients essential for sustaining human life, such as carbohydrates, fats, proteins, and fiber, and how they affect the human body. Nutrition studies mainly determine nutritional intake and status, correct eating habits, and supply nutrients to the entire population (1). Conversely, personalized nutrition begins with the concept that the occurrence of diseases or the degree of health maintenance owing to food intake may vary based on individual characteristics (2, 3).

Personalized nutrition is increasingly gaining attention in clinical settings, with healthcare professionals using genetic testing and other personalized approaches to manage chronic diseases and improve patient outcomes. With the advancement in science and technology and increased awareness, it is widely acknowledged that unhealthy eating habits, including overnutrition, cause various diseases (4–6). These diseases can be prevented by correcting unhealthy eating and lifestyle habits (7). An interesting recent report demonstrated that individualised and tailored nutrition for patients improves survival rates compared to conventional nutrition for the entire population (8, 9). Patients and the general public are receiving more attention as the social environment that values the individual is accelerating, and interest in health is increasing owing to the coronavirus pandemic.

Even if the same diet is provided, different phenotypes appear owing to differences in individual metabolic reactivity; furthermore, individual genetic diversity has been reported to cause various phenotypes according to nutrient intake (10). For example, a Dutch study on older adults found that providing personalized advice based on dietary, genetic, and physiological information reduced body fat percentage (11). Furthermore, clinical trials are currently underway to determine the possibility of controlling blood sugar levels based on personal microbiome information (12, 13). Furthermore, investigations are being conducted to ascertain the statistical significance of the correlation among the genes, diet, and disease of an individual and to evaluate the efficacy of dietary interventions in disease prevention (14, 15). In addition to lifestyle and eating habits, studies are underway to provide optimal personalized nutrition by analysing correlations based on data from individual intestinal microbes and discovering disease-related diagnostic indicators (16).

The field of personalized nutrition is being developed into numerous programs and apps to provide personalized nutrition and diet through the convergence of information and communication technology. Various methods have been implemented, such as professional data input, result input, and health checkup result input. Through this, individuals can manage their health by directly improving their eating habits, and it is expected that there will be an effect on preventing diseases that may occur in the future. Furthermore, it is important to develop algorithms and predictive models that combine genetic, clinical, and lifestyle data, including factors such as age, sex, activity level, and health status, to create personalized diet plans (17). Notably, wearable devices and mobile applications can monitor an individual’s diet, physical activity, and health metrics in real-time and use these data to provide feedback and personalized nutritional advice (18). Advanced computational techniques, such as artificial intelligence and machine learning, are being developed to analyse massive amounts of data and identify patterns for personalized nutrition recommendations (19).

However, the relationship between dietary intake and various individual characteristics, including genetic information, has not been fully elucidated, and its benefits have not yet been established in randomised controlled trials. Nevertheless, scientific verification and accuracy have not been achieved, necessitating further research results.

## 2 Nutritional genomics

Human health and life expectancy are influenced by a blend of genetic and environmental factors. Notably, significant efforts have been undertaken to mitigate these factors that are beyond human control. With the completion of the Human Genome Project in 2003, the genetic sequence of humans was fully revealed, and information on genetic polymorphisms has been continuously updated to understand individual characteristics and disease pathogenesis (20). Genetic polymorphisms influence characteristics between individuals; in particular, single nucleotide polymorphisms (SNPs) and differences in a single base pair in the DNA sequence are known to be highly associated with the pathogenesis of specific diseases (21–24); therefore, they are used as markers for predicting and diagnosing specific diseases, such as Alzheimer’s and celiac diseases (23, 25, 26). Furthermore, the overall exploration of human genomics has been accelerated by elucidating the correlation between the human microbiome, the genome of the human microbial community, and disease. These findings form the basis for personalized medicine, and related studies are steadily developing.

Food and nutrition are critical factors in maintaining health and disease onset. Furthermore, the development of genomics has affected the food and nutrition fields, raising the need for changes in dietary intake according to individual gene expression patterns. Consequently, the possibility of personalized precision nutrition has emerged. Therefore, because genomics is widely employed in the medical field, various attempts have been made to study the effects of food on the body by combining nutrition and genomics, referred to as nutritional genomics.

### 2.1 Nutrigenetics-genetic variation and nutrients metabolism

Specifically, nutritional genomics (nutrigenomics) studies the effects of the interaction between food (nutrients) and genes on the human body. It is classified into nutrigenetics and nutrigenomics (10).

Nutrigenetics aims to study the effects of genetic variants on food absorption and metabolism. Notably, SNP information from genome-wide association studies is the main source for studying differences in food responses to SNP (27–29). A representative example of nutrigenetics is the association between folic acid and 5,10-methylenetetrahydrofolate (MTHFR). The catalytic action of MTHFR metabolises folic acid, and genetic variations in MTHFR do not activate metabolism, leading to folic acid deficiency. The reported genetic variants of MTHR are C677T and A1298C (30, 31). Because the methyl group produced by folic acid metabolism is involved in the homocysteine (Hcy) remethylation pathway, in the case of an MTHR genetic variant, Hcy is not metabolised and accumulates in the body (32, 33). Increased blood Hcy concentration is a major risk factor for cardiovascular and cerebrovascular diseases. Therefore, in this case, sufficient folic acid supplementation is needed to compensate for genetic defects (34, 35).

### 2.2 Nutrigenomics-nutrients and epigenetic modification

Nutrigenomics has been developed using various omics technologies based on molecular biology (28, 36, 37); furthermore, related research fields are rapidly growing with the development of high-throughput technologies, such as microarrays (38). The goal of nutrigenomics is to study the effects of food (nutrients) on gene expression. Environmental factors, such as dietary intake, do not change the DNA sequence but contribute to changes in the phenotype by being involved in gene replication, transcription, and protein expression processes (10, 39).

Various studies have been conducted on epigenetic regulation, including DNA methylation, histone acetylation, and chromatin remodelling. In the case of diet-derived diseases such as obesity and diabetes, several CpG sites where DNA methylation occurs have already been found (40, 41). Importantly, methionine- and folate-containing methyl donors are representative food substances involved in DNA methylation. Interestingly, erythrocyte folate levels were lower in patients with type 2 diabetes (T2D) than in healthy participants; additionally, hypomethylation was observed at their hepatocyte CpG sites of T2D-related genes (42). Furthermore, it has been reported that DNA methylation at the CpG site exhibits different patterns according to the intake of saturated and unsaturated fatty acids. Additionally, histone acetyltransferase activity of pancreatic β cells is increased by the intake of saturated fatty acids (43, 44).

Furthermore, dietary components affect miRNAs, regulators that control gene expression. Specifically, polyphenols such as curcumin, resveratrol, and quercetin are known to regulate the expression of miRNAs in cancer cells and are involved in inflammatory responses (45). Additionally, the human gut microbiome is essential in personalized nutrition, as it plays a vital role in health, including digestion, nutrients, and absorption of food; however, it plays a similarly important role in overall health and research on personalized nutrition. In particular, research on personalized nutrition focuses on individual gut microbiome composition and its dietary response (46, 47).

### 2.3 Nutrition and gut microbiome

In the field of personalized nutrition, a highly regarded focus in recent years has been the interaction between nutrients and the microbiome. The research findings published in the 2006 issue of Nature, indicating a correlation between gut microbiota and obesity, played a pivotal role in fostering a new understanding of the microbiome’s significance (48). Since then, numerous studies have explored the interaction between the microbiome and health, revealing their effects on almost every metabolism in the body, including gut health and the immune system (49–51). In addition, unlike genetic information that remains unchanged once determined, the microbiome can change according to environmental factors such as exposure to food, drugs, and toxins, which can be attractive factors with epigenomics regarding disease prevention and health care. The increase in beneficial intestinal microbiomes, such as *Bifidobacterium* and *Firmicutes*, resulting from the consumption of prebiotics and probiotics exemplifies how symptoms such as constipation, obesity, and inflammatory responses can be alleviated (52–54). The research findings presented by the Wiseman Institute regarding the clinical efficacy of personalized nutrition marked a groundbreaking departure from conventional notions surrounding dietary approaches. This study indicates that individual variations in postprandial blood glucose responses to food exist. A personalized diet was developed using a machine-learning algorithm by integrating information on individuals’ blood parameters, dietary habits, anthropometrics, physical activity, and gut microbiota. Validation of this approach demonstrated an effective regulation of postprandial blood glucose and observable changes in gut microbiota (55). Based on these research findings, the demand for personalized nutrition has increased, with particular attention being focused on changes in the microbiome.

## 3 Core patents for personalized nutrition technology based on genomics

**Figure 1:**
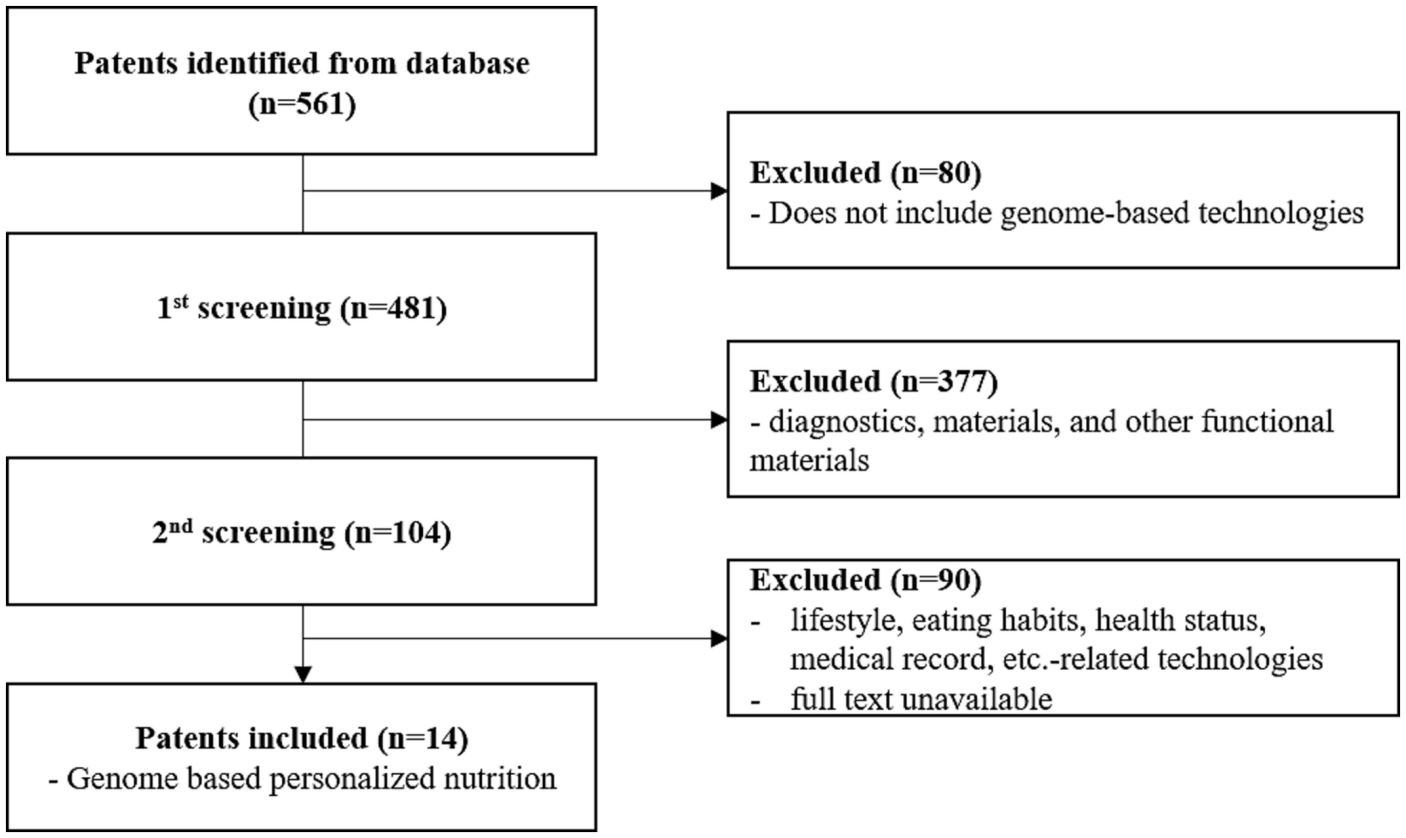
Patent selection flowchart.

**Figure 2:**
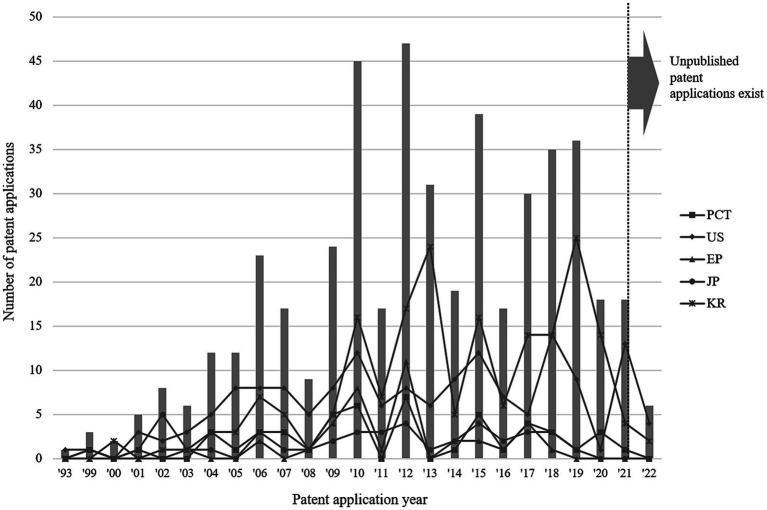
Patent trends related to genome-based utilization technology.

**Figure 3:**
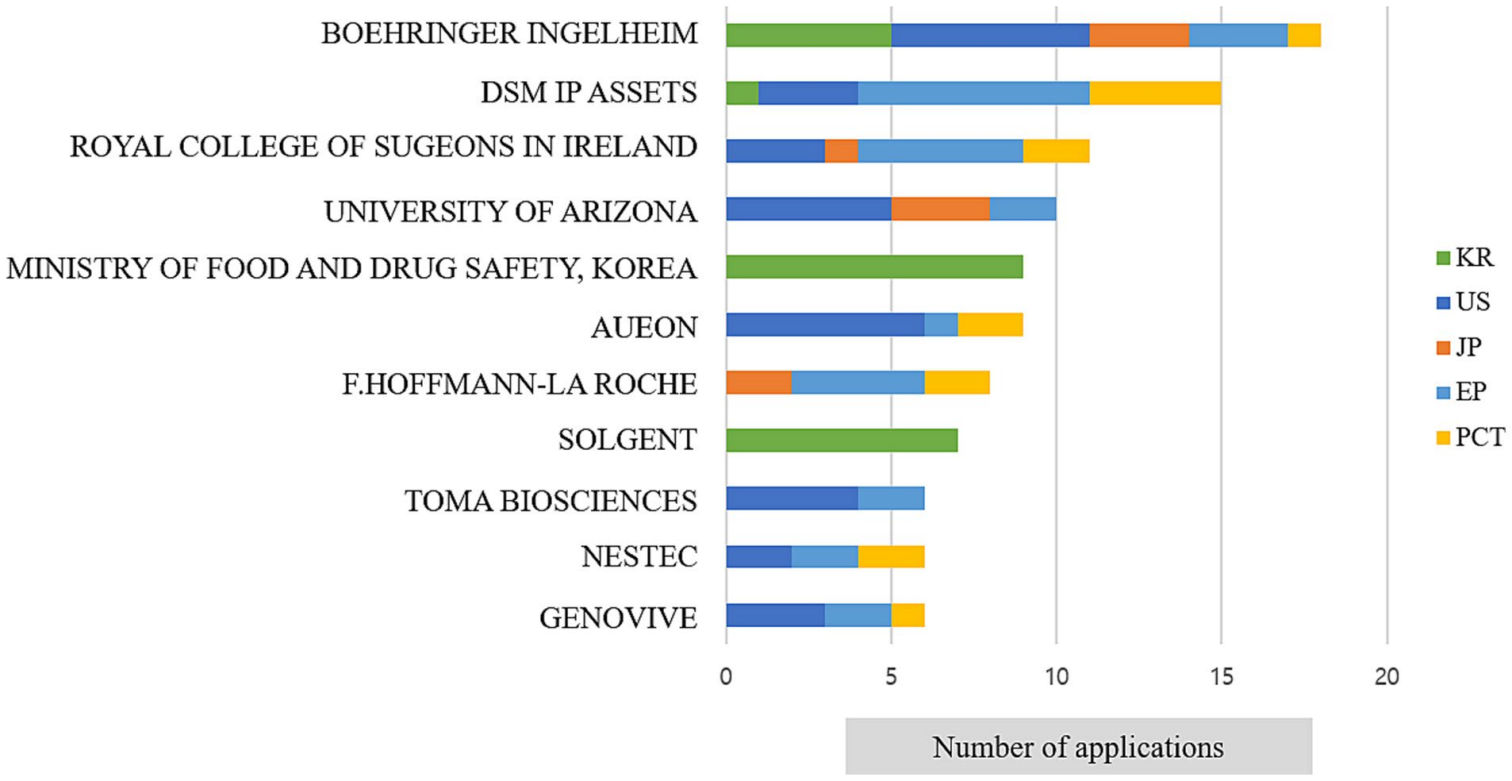
Major applicants for recommended technologies based on genome information.

To analyse genome-based personalized nutrition technologies, patents published and registered by December 2022 in Korea, Japan, the USA, Europe, and The Patent Cooperation Treaty countries were searched using data from World Intellectual Property Organization member countries. Search terms, including “nutrigenetic”, “nutrigenomic”, “epigenetic”, “single nucleotide polymorphism”, “DNA”, “metylat”, “acetylat”, “nutri”, “food”, and “diet”, were used to select relevant patents. Overall, 694 patents were searched, and 561 were selected based on highly relevant valid data (Table 1). Patent classification by technology was conducted for 561 valid patents, and trend analysis was conducted on 481 patents, excluding patents related to functional materials that did not include genomics. Among the valid data, 104 patents were selected, excluding diagnostics, materials, and other functional materials. Ultimately, 14 patents were selected for genome-based health care and personalized nutrition, excluding personalized nutrition technologies based on lifestyle, eating habits, age, health status, medical record, allergies, and patents with undisclosed full text (Figure 1 and Table 2). As shown in Figure 2, patents for genome-based utilization technology steadily increased from 1993 to the late 2000s and repeatedly increased and decreased from 2020 to 2022. Unpublished applications have existed since 2021, and the number of applications will likely increase further. This increase is owing to the acceleration of research, as the introduction of NGS technology in the mid-2000s made it possible to analyse genetic big data (56). As shown in Figure 3, the main applicants for the recommended technologies based on genome information were companies from various countries, including Boehringer Ingelheim. Full manuscripts were reviewed after examining the titles and abstracts, and relevant information and data were collected. References were examined for further relevant titles and literature and then reviewed.

**Table 1:** Valid patent criteria.

Main category	Subdivision	Number
Recommendation technology using genome information	Personalized nutrition technology	104
	Personalized drug technology	108
Diagnostic technology using genome information	diagnostic technology	217
Regulatory materials for genome	Materials for Genome expression or suppression	52
Functional materials	Other functional materials	80
Total		561

**Table 2:** Core patents for personalized nutrition technology.

No.	Publication or patent number	Applicant	Document title	Ref.
1	WO2006050939A1	DSM IP ASSETS B.V	System and method to compound a personalized nutritional supplement	(57)
2	US20120295256A1	GENOVIVE LLC	Weight management genetic test systems and methods	(58)
3	KR102144088B1	SOLGENT	Method of providing recommended dietary information for young child based on genetic test and system for the same method	(59)
4	WO2023094423A1	SOCIÉTÉ DES PRODUITS NESTLÉ S.A.	Personalized recommended daily intake for nutrients based on individual genetic risk scores	(60)
5	US11264129B2	XYGENYX INC	Selection and distribution of dietary supplement products based on DNA testing	(61)
6	WO2011064352A1	BOEHRINGER INGELHEIM INTERNATIONAL GMBH	Treatment of genotyped diabetic patients with DPP-iv inhibitors such as linagliptin	(62)
7	KR101540647B1	MINISTRY OF FOOD AND DRUG SAFETY, KOREA	Simultaneous multiple analysis of korean pharmacogenetic genotype for personalized medicine and methods for predicting drug response using diagnostic results	(63)
8	KR100844387B1	KOREA INSTITUTE OF ORIENTAL MEDICINE	UCP-2 and UCP-3 polymorphic markers for predicting body weight control of an obesity patient through low calorie dietetic treatment	(64)
9	US10966949B2	PARTHENOGEN SAGL	Dietary supplementation to achieve oxy-redox homeostasis and epigenetic stability	(65)
10	US11315686B2	Vivante Health, Inc.	Individualised care management system based on digestive activity	(66)
11	KR2298350B1	Ami cosmetic. Co., Ltd.	Method and device for providing customized health functional food manufacturing and recommendation information using microbiome	(67)
12	KR2278646B1	D.iF, Inc.	Customized food recommendation system	(68)
13	US9554754B2	Newtopia Inc.	System, method and computer program for weight, lifestyle and disease management integrating nutrition, exercise and behaviour management	(69)
14	EP1794316B1	Hill’s Pet Nutrition Inc.	Genome-based diet design	(70)

DSM IP Assets B. V reported a device for personalized nutritional supplements characterised by transmission to an automatic synthesis system; the input device receives information and transmits it to the control device (57). Specifically, a device for automatically preparing nutritional supplements tailored to the specific genotype, phenotype, or personal preference of a participant was characterised by analysing DNA samples for a subset of genes from the participant’s blood, urine, and hair samples. Furthermore, the food intake and physical activity levels of an individual are evaluated to prepare personalized nutritional supplements (ingested food types, intake amount, alcohol and caffeine consumption, and personal information such as sex, height, and weight). Based on the results of the genotype and phenotype analyses, a personal nutritional supplement recipe is prepared using an algorithm.

Genovive L.L.C. has published a patent on personalized nutrition that includes a weight management genetic testing system and a method for individualised diet and exercise programs using DNA testing for specific genes and genetic variants (58). The system analyses the DNA test kit of the customer (SNP analysis system) to diagnose genetic variants that strongly affect weight management and recommends a diet or exercise method based on the cumulative score for the genetic variants using an algorithm. Using the weight, height, waist and hip circumference, body mass index, waist-to-hip ratio, gender, and racial characteristics, genetic variations among rs1047214, rs1799883, rs1800588, rs1800629, rs1800795, rs1801282, rs2070895, rs4343, rs4994, rs5082, rs1042713, rs9939609, and rs294364,1are determined. Subsequently, a tailored diet and exercise plan are selected for the customer.

Solgent has registered a patent for a method and system that provides recommended dietary information to children based on genetic testing. Specifically, it provides food or nutritional information to improve autism or developmental delays using genetic information and systems (59). During genetic diagnosis, the system determines the gene sequence to be tested from a biological sample of the child. Next, the DNA sequence determined through the gene sequence inputter and converter is converted into a suitable file format. The reference and boundary sequence databases are compared with the gene to be tested to determine whether there is a base mutation at the SNP site. A database is employed that focuses on the improvement or prevention of autism or developmental delays to offer information on food or nutrients. Specifically, this database is associated with the SNP base information of the gene to be tested, which is related to the methylation cycle.

Société des Produits Nestlé S.A. has published a patent for personalized recommended daily nutrient intake based on individual genetic risk scores (60). For genetic diagnosis, the SNP genotype profile of the DNA sample of the user is compared with that of the reference SNP. A genetic risk score for each nutrient for at least one plurality of nutrients is calculated to provide intake information. Based on the risk score, a personalized recommended daily intake is provided after comparison with the recommended daily intake for each nutrient.

Xygenyx INC. has registered a system for selecting and distributing dietary supplements based on DNA test results (61). For genetic diagnosis, samples are taken from users using gene kits and analysed. After identifying the dietary supplements beneficial to user health based on the genetic test results of the user through an algorithm, the amount of dietary supplements is determined and provided according to body information (age and weight). Specifically, as a selection and distribution system for health supplements based on genetic testing, the server includes a non-volatile data storage device and health supplement data. Data, including the name, age, weight, health status, and genetic profile of the client, are collected and stored in a database. Subsequently, dietary supplements that are known or suspected to be beneficial to a health condition are identified, and the amount of dietary supplements is determined based on the age and weight of the customer. Finally, recommendations are sent to customers.

Boehringer Ingelheim International GmbH has registered patents to provide agents and methods for preventing, delaying, and treating metabolic disorders, particularly type 2 diabetes (62). This is particularly applicable to patients with one or more mutations in TCF7L2, known to elevate the incidence and progression of type 2 diabetes. Patients undergo testing for specific SNPs (e.g., an SNP selected from rs7903146, rs12255372, or rs10885406) in the gene encoding TCF7L2. After diagnosis, patients with the TCF7L2 genotype receive prescriptions for linagliptin, a DDP-4 inhibitor, which can be optionally administered in combination with other antidiabetic drugs such as biguanides, thiazolidinediones, sulfonylureas, glinides, alpha-glucosidase inhibitors, and GLP-1.

The Ministry of Food and Drug Safety of the Republic of Korea has registered a patent for a drug response prediction method that uses simultaneous multiplex analysis of Korean drug genotypes and results for applying personalized drugs (63). By integrating genes related to drug effects, adverse reaction prediction, and pharmacokinetic response, numerous drug genotypes can be analysed through an optimal method capable of handling large volumes and predicting individual drug responses based on genotype analysis results. Specifically, this drug response prediction approach employs multiple simultaneous analyses of drug genotypes and their diagnostic outcomes. Genetic diagnosis is performed to select drug genes and genotypes affecting drug effects, adverse reaction prediction, and pharmacokinetic responses in Korean individuals and construct analysable combinations. Using genomic DNA isolated from the subjects, possible combinations (glass microbead assay method and SNaPshot method) are constructed for large-scale analysis of drug effects, adverse reaction prediction, and pharmacokinetic response-related genes and genotypes. The drug response related to the drug genotype can be predicted in advance by predicting the biological activity of the individual using the drug genotype diagnosis results obtained using the analysis method.

The Korea Institute of Oriental Medicine has applied for a patent for a polymorphic marker for predicting weight control related to obesity and fat burning (64). It was claimed that coupling protein-2 (UCP-2) and uncoupling protein-3 polymorphism markers associated with diet-induced weight change in obese patients can be used. Looking at the contents, genomic DNA is isolated from a blood sample of a test subject for genetic diagnosis, and polymorphic sites present on genomic DNA are detected using a kit for predicting weight control. Furthermore, weight control is achieved by analysing the correlation between the detected polymorphic region and alterations in the obesity phenotype following a low-calorie diet. Notably, the marker consists of a polynucleotide comprising 20–100 consecutive DNA sequences, which include the -866th base from the transcription start point of the UCP-2 gene (the 63rd sequence of SEQ ID NO:2 is G) and the -866th base sequence. Specifically, this is identified as a UCP-2-866G > A polymorphic marker.

Parthenogen is registered as a dietary supplement aimed at achieving oxy-redox homeostasis and ensuring epigenetic stability (65). Specifically, it acts as a micronutrient to achieve effective oxy-redox homeostasis with improved energy balance, cell growth, and differentiation processes, including DNA methylation and epigenetic regulation. Technical features include methyl tetrahydrofolate and its pharmaceutically acceptable salts in the range of 100 to 800 μg folic acid equivalents, as well as methylcobalamin and its pharmaceutically acceptable salts in a quantity between 0.5 and 10 μg cyanocobalamin equivalents. Furthermore, the formulation includes at least two B vitamins selected from vitamin B2, nicotinamide, vitamin B3, and vitamin B6 and their pharmaceutically acceptable salts in an amount ranging from 0.4 μg to 40 mg. Betaine is present in a quantity between 100 and 2000 mg and/or its pharmaceutically acceptable salt. Notably, the compositions comprise 5–50 mg of a zinc compound and 100–2000 mg of a cysteine derivative without incorporating any antioxidants.

Vivante Health, Inc. has registered a patent for a personalized care management system based on digestive activity (67). This system measures the methane or hydrogen level emitted from the patient’s breath or stool samples, communicates this to a database through a device, and provides personalized dietary guidance. The core technology involves a system that communicates with a hydrogen or methane sensor device based on the gut microbiome and digestive activity. Its analysis uses bioinformatics to detect changes in hydrogen and methane levels based on an individual’s gut microbiome and dietary intake. The inventor has published a process for intestinal microorganism analysis wherein stool samples are collected. Various bacterial populations are identified and quantified, and the microbiome results are compared with healthy gut microbiome information to identify factors causing the client’s chronic digestive disease symptoms. Additionally, the inventors disclosed a procedure for analysing gut microorganisms, including a stool sampling kit, a stool sample container, an optional expandable stool culture bag, an analysis package, and an impermeable bag for air and liquid. Bioinformation and its relationship to changes in hydrogen and methane levels are also used to infer drug effects.

Ami cosmetic. Co., Ltd. has registered the method and device for providing customized health functional food manufacturing and recommendation information using microbiome analysis to determine the intestinal situation of an individual’s microbiome analysis, and the individual’s genetic analysis results are independently obtained (68). It can determine the priorities of nutrients suitable for the individual through intestinal situation and genetic analysis, and provide information on the manufacture and recommendation of health functional foods containing food ingredients.

D.iF, Inc. has registered a customized food recommendation system that recommends foods suitable for an individual by combining various information, including genetic information (69). This includes food metabolite information matching food identification data, relationship details indicating gene activation or suppression for each trait, and one or more of the customer’s genetic, physical, health status, and life log information.

Newtopia Inc. has registered the system, method, and computer program for weight, lifestyle and disease management, integrating nutrition, exercise, and behaviour management (70). This technology offers personalized disease management system that can identify, analyse, and classify personal attributes and suggest optimal solutions. For example, weight control involves analysing an individual’s nutritional suitability, behavioral characteristics, and obesity-related genes FTO, MC4R, and DRD2 genes. Through this analysis, classification and templates optimized for the individual can be defined and provided.

Hill’s Pet Nutrition Inc. has registered a genome-based diet design, a method that provides nutrition to animals by identifying breed clusters through genome analysis and selecting animal food with a nutritional composition that matches the animal’s nutritional needs according to breed characteristics (56). This technology also involves preparing food by mixing bioactive dietary ingredients in amounts and proportions consistent with the nutritional formula. Bioactive dietary ingredients are substances that promote the health of animals and include nutrients and substances essential for life. These ingredients generally include naturally occurring chemical substances found in certain plants and foods and those manufactured through microbiological (e.g., fermentation) or synthetic processes. The process of identifying the animal’s characteristics involves finding SNP characteristics and distinguishing between different breeds through genetic analysis of biological fluids such as excrement, blood, saliva, and amniotic fluid.

## 4 Discussion

As a result of technology growth analysis based on patent analysis, examinations are being conducted on diagnostic and recommendation technologies using genome information. Research and development of recommendation technologies utilising genome information continue worldwide, and studies on genome control materials are rapidly expanding. In the future, it is anticipated that research on genome control materials will be further invigorated alongside the recommended technologies for leveraging genome information. Recommendation technologies that use genomic information can be divided into personalized nutrition and drug recommendation technologies. The results of a patent review revealed that personalized drug technology accounts for a greater share of personalized nutrition technology, reflecting the urgency of the demand for medicines that can treat diseases. Accumulated research results and databases on disease-related genomes and various related markers and compounds are well-secured, which seems to have promoted the development of personalized drug technology. Furthermore, the development of genome analysis technology has led to the expansion of technology investment by global diagnostic and pharmaceutical companies to secure personalized drug technology. Drug recommendation technology that utilises genetic information has been recognised as a technology that offers personalized drug suggestions to patients. Importantly, this is based on results obtained from establishing a correlation between a patient with a specific disease and the corresponding gene for that disease through experimental studies.

Furthermore, experiments have demonstrated that diagnostic technology employing genetic information and gene control materials also holds promise for diagnosis or materials following the revelation of correlations between specific diseases and genes. Interestingly, compared to the past, patent applications for drug recommendation technologies show a stagnant or decreasing trend. In recent years, research and development in personalized nutrition based on genetic information have been actively underway, extending beyond the medical aspect. With the emergence of nutritional genomics, where individual responses to food intake change based on genetic information and gene expression varies with nutrient intake, related technologies are on the rise. Specifically, epigenomics and the microbiome, influenced by external factors and exhibiting individual variations, are major contributors to the growth of personalized nutrition. In the context of nutrition recommendation technologies using genetic information as presented through various patent information, it is assessed that a technology that scores individual genes and body information using a pre-identified genetic database or recommends a diet specific to a certain disease is registered. From the analysis of the registration history for each technology, it is expected that personalized nutrition technology using genetic information will develop in the direction of deriving correlations between specific foods, diseases, and genes, similar to drug recommendation technology using genetic information. Therefore, to increase the possibility of securing technology in the field of personalized nutrition using genetic information, it is desirable to derive the relationship between a specific gene and nutrition using experiments and use the results to secure a recommendation technology. Nevertheless, it has been confirmed that the application of personalized nutrition technology is steadily increasing, and it is expected that patent applications for related technologies will continue owing to this increasing trend.

## 5 Conclusion and future perspective

In this review, we comprehensively analysed patent information containing technologies related to personalized nutrition based on genomics. Through various patent reviews, it could be predicted that recommendation technologies using genomic information will continue to increase in the field of personalized nutrition. However, validating these technologies through experiments is necessary for more practical application.

## Author contributions

S-HP: Data curation, Investigation, Writing – original draft, Writing – review & editing. H-KC: Writing – original draft. JP: Supervision, Writing – original draft, Writing – review & editing. J-TH: Conceptualization, Data curation, Supervision, Writing – original draft, Writing – review & editing.
